# HDDF2—A Novel Patient-Derived Fibroblast Line from Huntington’s Disease with Prominent Cellular Senescence and polyQ Pathology

**DOI:** 10.3390/biomedicines14071484

**Published:** 2026-06-30

**Authors:** Polina Parfenova, Nina Kraskovskaya, Anna Koltsova, Alla Shatrova, Natalia Yartseva, Natalia Mikhailova

**Affiliations:** Institute of Cytology Russian Academy of Science, 194064 Saint-Petersburg, Russia; sucrederaisin@incras.ru (P.P.); koltsova.am@mail.ru (A.K.); shatrova@mail.ru (A.S.); ya.ya-natm27951@yandex.ru (N.Y.); natmik@mail.ru (N.M.)

**Keywords:** fibroblasts, cell line, Huntington’s disease, huntingtin, polyQ, direct reprogramming

## Abstract

**Background/Objectives**: Patient-derived cell lines retaining donor-specific age-related and genomic features are essential for modeling late-onset neurodegenerative disorders like Huntington’s disease (HD). This study aims to establish and comprehensively characterize HDDF2, a novel dermal fibroblast line from an HD patient, to provide a relevant cellular model. **Methods**: Dermal fibroblasts were isolated and cultured from a 44-year-old male HD patient carrying 46 CAG repeats in the *HTT* gene. Cells were evaluated for senescence markers (p16, lamin B1, SA-β-Gal activity, proliferation rates) and polyglutamine (polyQ) aggregation. Direct reprogramming protocols were applied to convert these fibroblasts into induced neurons. **Results**: HDDF2 fibroblasts exhibited a pronounced senescence-associated phenotype, evidenced by increased p16 expression, reduced lamin B1 levels, elevated SA-β-Gal activity, and decreased proliferation. Notably, polyQ deposition was preferentially detected within the senescent subpopulation, displaying distinct localization patterns differentiating senescent from proliferating cells. Despite this, HDDF2 cells retained their capacity for direct reprogramming and were successfully converted into induced neurons. **Conclusions**: HDDF2 represents a well-characterized, patient-specific cellular model for HD. The observed co-occurrence of polyQ deposition and cellular senescence, combined with successful neuronal conversion, establishes this line as a valuable resource for investigating the relationship between cellular aging and HD pathogenesis.

## 1. Introduction

Huntington’s disease (HD) is a rare and incurable neurodegenerative disorder that is inherited in an autosomal dominant pattern, with near-complete penetrance in most cases. It is caused by a mutation in the huntingtin gene (*HTT*), which leads to the expansion of CAG repeats in exon 1 beyond a threshold of 36 codons. This causes the production of mutant huntingtin (mHTT) protein, which triggers a cascade of neurotoxic events and leads to the development of the disease. Clinically, HD most commonly manifests between the ages of 30 and 50, though rarer and more severe juvenile forms can present in childhood or adolescence [1].

At the molecular level, the hallmark of HD progression is the accumulation of cytoplasmic mHTT aggregates in the striatal neurons [2,3,4,5,6]. This phenomenon is evident in post-mortem tissues, as well as *in vivo* and *in vitro* models of HD [7,8,9,10]. The aggregated form of mHTT is believed to disrupt intracellular metabolic and transport processes through direct interaction with various intracellular proteins [11,12,13]. The initial investigations into HD were performed using animal models, specifically mice, which allowed researchers to study the molecular mechanisms of the disease and identify potential targets for treatment [14,15,16,17]. In addition, modeling of the disease in a diverse range of animal species at various levels of biological organization has facilitated the investigation of the fundamental molecular mechanisms of huntingtin function. This approach has allowed us to, for example, discover the widespread presence of *HTT* and its homologs in a variety of organisms, including *Caenorhabditis elegans*, *Drosophila melanogaster*, and even sea urchins [18,19,20]. Later animal models were extended to other larger animals, including minipigs, sheep, and non-human primates [21,22,23]. Although these investigations deepened knowledge of the mHTT functioning, they do not fully reproduce key aspects of human polyglutamine-dependent pathology, including the precise cellular context and the role of human-specific genetic modifiers. This is supported by numerous successful studies of potential drugs on animals that, unfortunately, failed in clinical trials [24,25,26]. Consequently, translating insights from rodent models to human patients remains a major challenge, limiting the development of effective therapies [17,27,28].

Recently, there has been active development of patient-specific cell models for HD. Initially, researchers used embryonic stem cells with a mutation in the *HTT* gene to model the disease, but these cells have ethical and legal limitations [29,30]. A significant breakthrough was the creation of induced pluripotent stem cells (iPSCs), which can be derived from a patient’s own somatic cells, such as fibroblasts, through reprogramming [31,32]. This method eliminates ethical concerns and provides an unlimited supply of cells for research. There are well-established protocols for the differentiation of iPSCs into medium spiny neurons (MSNs), which are the target cells in HD pathology [9,33,34]. However, the process of generating iPSCs and differentiating them can be time-consuming, and it may also neutralize age-related epigenetic changes that are important for pathogenesis [35,36].

An alternative approach is the direct conversion of patient somatic cells into MSNs, bypassing the pluripotent stage. This method preserves the epigenetic profile and age-related characteristics of donor cells, making the model more suitable for studying late-stage neurodegenerative diseases like HD [37,38,39,40]. Therefore, current HD research combines the use of traditional animal models with promising patient-specific cellular systems. This allows for a more comprehensive understanding of the molecular mechanisms underlying the disease and the testing of new therapeutic strategies. Thus, dermal fibroblasts represent a crucial starting material for creating such models. Despite their importance, there is a lack of readily available and fully characterized fibroblast cell lines from HD patients that have been documented to maintain disease-relevant phenotypic features and proven to be capable of neuronal reprogramming. To address this gap, we present the detailed molecular biological, cytogenetic, and functional characterization of a new dermal fibroblast cell line (HDDF2) derived from a patient with HD. We also demonstrate its suitability as a starting material for direct reprogramming into neuronal cells, making this line a valuable tool for pathogenetic research and therapeutic agent screening.

## 2. Materials and Methods

### 2.1. Cell Isolation from Skin Biopsy

All procedures involving biological material were performed after obtaining approval from the ethics committee (protocol of the bioethics commission dated 8 August 2023) and informed consent from the donor. A skin fragment was obtained from a 44-year-old patient with HD, who had 46 CAG repeats, and then it was mechanically dispersed with a sterile scalpel into a 1 mm^2^ fragment and placed in a Petri dish under a sterile glass coverslip. The fragments were cultured in 90% DMEM (Macklin, Shanghai, China) with 10% fetal bovine serum (LT Biotech, Vilnius, Lithuania) at standard conditions: 37 °C, 5% CO_2_, 95% humidity. When the cells reached 80% monolayer density, they were passaged with a solution containing 0.25% trypsin (Biolot, Saint-Petersburg, Russia) and 0.2% Versene solution (Biolot, Saint-Petersburg, Russia) in a 1:2–1:3 ratio. It is important to note that the cultivation process was conducted without the use of antibiotics or antifungal medications. This allowed for regular visual inspection using a light microscope to monitor bacterial and fungal contamination. Additionally, two methods were used to detect mycoplasma infection: direct fluorescent staining of DNA with Hoechst 33258 dye (Sigma-Aldrich, St. Louis, MO, USA) and seeding on selective nutrient media. These procedures were carried out in accordance with previously described methods [41,42]. The uniqueness of the new cell line and absence of cross-contamination with other cell lines were determined using fragment analysis of human STR markers.

Additionally, the dermal fibroblast line HdFb220 (healthy male donor, 41 years; Koltsov’s Institute of Developmental Biology, Russian Academy of Sciences, Moscow, Russia) was used as a non-HD control in immunofluorescence experiments and was maintained under identical culture conditions.

### 2.2. Flow Cytometry

#### Growth Curve

Cell viability was assessed by excluding DAPI-positive (dead) cells, while the total number of DAPI-negative cells was used as a proxy for proliferative activity. Staining was performed immediately prior to flow cytometric analysis on CytoFLEX and CytoFLEX S (Beckman Coulter, Brea, CA, USA). Data were collected and processed using the CytExpert software (version 2.3). Cell counts were collected for viable cells (DAPI-negative). The experiment consisted of five time points (0, 24, 96, 168, and 240 h) with three technical replicates per time point. Cells were seeded at a density of 5500 cells/cm^2^ at passage 6. Based on the obtained data, the relative proliferation index (PI) was calculated as the ratio of the number of DAPI-negative cells at each experimental time point to that at the zero time point.

The average cell population doubling time was calculated using the following formula [43]:

$$a_{0}=(t\cdot ln2)/(ln(M_{t}⁄M_{0})),$$

where t  is the time of the logarithmic growth phase, $M_{t}\;$ is the cell number at the point where the growth curve reaches a plateau (t), and $M_{0}$ is the cell number at the beginning of the logarithmic growth phase.

### 2.3. Immunophenotyping

Prepared cells (concentration of 1 × 10^6^ cells per mL in staining buffer) were incubated with a panel of fluorescently labeled antibodies: PE-conjugates against CD34, CD44, CD73, CD90, and CD105, as well as FITC-conjugates against HLA-ABC. Nonspecific binding was controlled using isotype antibodies (Table 1). All procedures were performed according to the manufacturer’s recommendations, and the data were analyzed using CytExpert 2.3 software.

### 2.4. Cloning Efficiency

Cells were seeded in Petri dishes at a density of 2–3 cells/cm^2^. Twelve dishes were prepared. After 14 days, cells were stained with 1% aqueous crystal violet solution, and colonies containing at least 20 cells were counted. Cloning efficiency per dish was calculated as (number of colonies/10 seeded cells) × 100%. Data are presented as median with interquartile range (Q1–Q3).

### 2.5. Cytogenetic Analysis

For cytogenetic analysis, cells were accumulated at the metaphase stage by incubation with colchicine (0.1 μg/mL; Sigma-Aldrich, USA) for 3 h. After the incubation period, cells were detached with a solution containing 0.25% trypsin (Biolot, Saint-Petersburg, Russia) and 0.2% Versene (Biolot, Saint-Petersburg, Russia). Then the cell suspension was sequentially hypotonicized with a mixture of 0.55% KCl and 1% sodium citrate (1:1, 15–20 min, 37 °C) and fixed with a cooled mixture of methanol and acetic acid (3:1, three times for 15 min). Metaphase plate preparations were made by drop-splitting onto glass slides over a water bath (50–53 °C). Chromosomes were stained using the standard GTG banding method: dried preparations were treated with 0.02% trypsin solution (Biolot, Saint-Petersburg, Russia) for 2–3 min, the reaction was stopped in a GKN solution (15 s), and counterstained with 2% Giemsa solution (Merck Millipore, Burlington, MA, USA) in phosphate buffer (4 min). Analysis of chromosomal rearrangements was performed automatically using a system based on an Axio Skop A1 ProgRes MF microscope (Carl Zeiss, Oberkochen, Germany), a CCD camera (Jenoptik, Jena, Germany), and VideoTest Karyo 3.1 software.

### 2.6. STR Profiling

Short tandem repeat (STR) profiling was performed for the identification of the HDDF2 cell line by 28 loci. STR loci and the amelogenin sex-determining marker were amplified using the “Gene Profile Human” Kit for 28 STR loci (Syntol, Moscow, Russia) (detecting amelogenin, D3S1358, D2S441, D1S1656, D19S433, TPOX, D2S1338, THO1, D16S539, CSF1PO, D18S51, D21S11, Yindel, D22S1045, D8S1179, VWA, D5S818, FGA, D13S317, D7S820, D12S391, D10S1248, SE33, DYS391, D8S1132, D7S1517, PENTA D and PENTA E) according to the manufacturer’s instructions in a T100™ Thermal Cycler (Bio-Rad, Hercules, CA, USA). Electrophoretic analysis was performed using a Nanophore 05 DNA Analyzer (Syntol, Moscow, Russia). After electrophoresis, the data were analyzed with Gene Marker^®^ HID Software v3.0.0 (SoftGenetics, LLC, State College, PA, USA) to categorize peaks by size in relation to an internal standard allelic ladder.

### 2.7. Assessment of Replicative Aging

The activity of senescence-associated β-galactosidase (SA-β-gal) was detected using a proprietary Senescence Galactosidase Staining Kit (Sigma-Aldrich, USA), following the manufacturer’s instructions. SA-β-gal activity was identified by the presence of blue-green granules within the cell cytoplasm. For quantitative analysis, images were captured, and a custom MATLAB (version 7.5.0.342 (R2007b)) script was used, based on the protocol by Shlush et al. [44]. To quantify the staining intensity per cell, the script described in the noted article above calculates the β-galactosidase Activity Value (BGAV). This was performed by selecting a region of interest (ROI) around a single cell and a corresponding background ROI. The Cell Staining Intensity (CSI) was calculated as the ratio of the sum of the blue and green channel pixel intensities to the sum of all three channels (RGB) within the cell ROI. The Background Staining Intensity (BSI) was calculated identically for the background ROI. The final BGAV for each cell was then calculated as the log10(CSI/BSI). SA-β-Gal positivity was quantified in a single experiment by scoring > 100 randomly selected, non-overlapping cells. Error bars represent 95% Wilson confidence intervals for proportions.

### 2.8. Immunofluorescent Staining

For immunofluorescence staining, fibroblasts were processed sequentially according to the following protocol. Cells were fixed in 10% formalin solution (Sigma-Aldrich, USA) for 20 min at room temperature, after washing with PBS. After a series of washes with PBS, membranes were permeabilized with 0.25% Triton X-100 (5 min, room temperature). Nonspecific binding was blocked with 3% bovine serum albumin (BSA, Sigma-Aldrich, USA) in PBS for 1 h. Cells were then incubated with primary antibodies diluted in blocking solution at +4 °C overnight. The following primary antibodies were used for immunocytochemical analysis: anti-β-tubulin III (R&D systems, Minneapolis, MN, USA, MAB1195, dilution 1/1000), anti-MAP2 (Abcam, Cambridge, UK, ab281588, 1/1000), anti-DARPP-32 (Thermofisher Scientific, Waltham, MA, USA, MA5-32113, 1/250), anti-GABA (Sigma Aldrich, A2052, 1/500), anti-mHTT (clone mEM48, Merck Millipore, MAB5374, 1/200), anti-polyQ (Merck Millipore, MAB1574, 1/1000), anti-p16 (Cell Signaling 18769, 1/500), anti-laminB1(Novus Bio, Centennial, CO, USA, NBP2-59783, 1/1000), anti-vimentin (Abclonal, Woburn, MA, USA, A19607, 1/2000), anti-GOLGA4 (p230) (Sigma Aldrich, HPA040675, 1/200). After washing off the primary antibodies, the cells were treated with fluorescently labeled secondary antibodies (45 min, room temperature). The following secondary antibodies conjugated with fluorophores were used for visualization: against mouse IgG—Alexa Fluor 555 (Abcam, ab150114, dilution 1:2000) and Alexa Fluor 488 (Abcam, ab150078, 1:2000); against rabbit IgG—Alexa Fluor 555 (Abcam, ab150113, 1:2000). The coverslips were washed three times with PBS and mounted on coverslips using mounting medium (Cell Signaling, Danvers, MA, USA). Images were acquired and analyzed using an Olympus Fluoview FV3000 laser confocal microscope (Tokyo, Japan).

### 2.9. Adipogenic, Chondrogenic and Osteogenic Differentiation

Adipogenic, osteogenic, and chondrogenic differentiation were induced to assess the status of mesenchymal stromal cells (MSCs). The cells were seeded at a concentration of 10,000 cells/cm^2^ in appropriate differentiation StemPro media (Gibco, Grand Island, NY, USA), and cultured for three weeks, changing the media every four days. After the incubation period, the cells were fixed with 10% formalin solution (Sigma-Aldrich, USA) for 15 min at room temperature and stained with appropriate dyes to detect the differentiations. For adipogenic differentiation, the cells were stained with Oil Red O Solution (Sigma-Aldrich, USA) for 15 min. To identify calcium salts that were formed during osteogenic differentiation, cells were stained for 15 min with an Alizarin Red Solution (Millipore, Shanghai, China). Chondrogenic differentiation was determined by the presence of sulfated glycosaminoglycans, which were revealed by staining with a 1% toluidine blue solution (MP Biomedicals, Irvine, CA, USA). Images were acquired with an AXIOVERT 200M microscope (Carl Zeiss, Jena, Germany) and a high-resolution color digital camera (DFC420, Leica, Wetzlar, Germany).

### 2.10. Direct Reprogramming into Induced Neurons

Direct reprogramming into induced striatal neurons was performed based on a published method [40] with the following optimization [45]. A confluent monolayer of HDDF2 fibroblasts (passage 10) in a 6-well plate was infected with lentiviruses carrying the microRNA genes MYT1L and CTIP2 and simultaneously treated with 1 μM rapamycin. After antibiotic selection, the cells were transferred to Matrigel-coated coverslips and additionally transduced with a lentivirus with DLX2. After 24 h, the medium was replaced with an induction medium (Neurobasal-A/B-27 with a cocktail of neurotrophins and small molecules: valproic acid, retinoic acid, dibutyryl-cAMP, BDNF, NT3). To complete reprogramming, cells were cultured for 35–40 days in induction medium in the presence of doxycycline 1 ug/mL final concentration.

### 2.11. HTT CAG Repeat Genotyping

Genomic DNA was extracted from HDDF2 fibroblasts at passage by phenol–chloroform extraction. CAG repeat length in exon 1 of the *HTT* gene was determined by fluorescent PCR followed by capillary electrophoresis (fragment analysis) performed twice by two independent companies (Labpack, Saint-Petersburg, Russia, and Syntol, Moscow, Russia). Briefly, the repeat-containing region was amplified using the primers [HD1F: 5′-ATGAAGGCCTTCGAGTCCCTCAAGTCCTTC-3′ (6-FAM) and HD3R: 5′-GGCGGCTGAGGAAGCTGAGGA-3′]. PCR products were analyzed on a Nanophor 05 (Syntol, Moscow, Russia). Repeat numbers were calculated from fragment sizes using GeneMarker (version 3.0.1) with an internal size standard S550 (COrDIS, Moscow, Russia).

### 2.12. Statistic

Quantitative data are presented as indicated for each experiment. SA-β-Gal fluorescence intensity data, which did not follow a normal distribution (Shapiro–Wilk test, *p* < 0.05), are presented as median [IQR]; no inferential statistical comparison was performed. Growth curve data are presented as mean ± SD of three technical replicates per time point. All analyses were performed using GraphPad Prism 10.6.1 (GraphPad Software Inc., Boston, MA, USA).

## 3. Results

**Figure 1:**
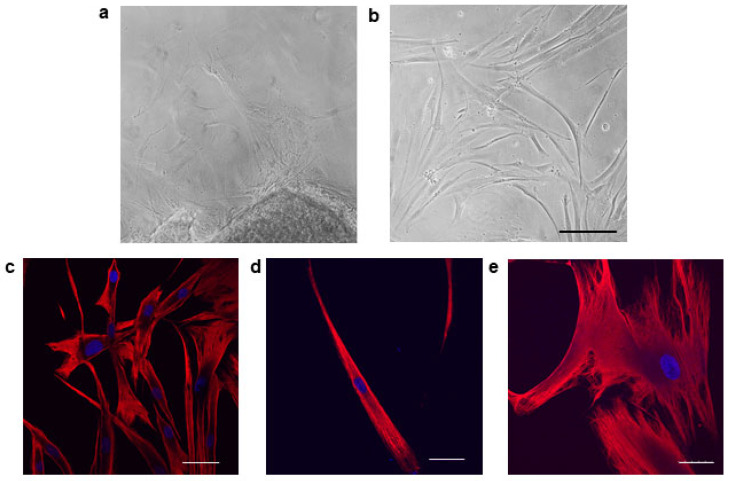
HDDF2 cell line morphology. (**a**). Isolation of dermal fibroblasts from a skin biopsy fragment by the migration method—3.5 weeks. (**b**). Morphology of the cell population at the 5th passage. Light microscopy, ×10; scale bar, 200 um. (**c**–**e**). Immunofluorescent staining of HDDF2 for DAPI and fibroblasts-specific protein vimentin. (**c**). Heterogeneous population. (**d**). Subpopulation of spindle-shaped appearance. (**e**). Subpopulation of enlarged and flattened appearance. Confocal microscopy, ×40; scale bar, 50 um.

In this study, we have successfully established and characterized a new dermal fibroblast cell line, designated HDDF2. The cell line was derived from a skin biopsy from a 44-year-old male patient with a genetically confirmed diagnosis of HD carrying 46 CAG repeats in the *mHTT* allele. The primary culture was established by mechanical disaggregation of tissue into small pieces, which were then cultured under standard conditions. Cellular growth from the explants was slow, with the first visible fibroblasts appearing only after a three-week incubation period (Figure 1a). The initial rate of proliferation was dependent on the number of migrating cells and their division activity, leading to a prolonged timeframe for establishing a confluent population suitable for the first passaging. After the initial slow expansion, cells began to proliferate more actively, and by passage five, a sufficient number was obtained for cryopreservation and subsequent experiments (Figure 1b). All detailed characterizations of the cell line were conducted between passages 6 and 10. Quality control analysis confirmed that the HDDF2 line was free from bacterial, fungal, and mycoplasma contamination. Short tandem repeat (STR) profiling confirmed the unique genetic identity of the line (amelogenin: X, Y; CSF1PO: 10, 13; D3S1358: 16, 19; D7S820: 12, 12; D5S818: 9, 13; D8S1179: 13,13; D13S317: 12, 13; D16S539: 9, 11; D18S51: 14, 15; D21S11: 28, 31.2; FGA: 24, 24; THO1: 7, 9.3; TPOX: 8, 9; vWA: 14, 19) and excluded cross-contamination with other cell lines. According to the requirements of International Cell Line Autentification Committee (ICLAC), 13 nuclear STR loci plus amelogenin to determine the sex are submitted. The full specific profiling results were not shared publicly due to patient identifiers. Morphological analysis revealed that the HDDF2 culture consisted of a heterogeneous population. To assess the cells’ morphology, immunofluorescent staining with vimentin antibodies was performed. Most cells had the typical spindle-shaped fibroblast morphology (Figure 1d). However, a significant subpopulation had an enlarged and flattened appearance, suggesting a senescent state (Figure 1e).

**Figure 2:**
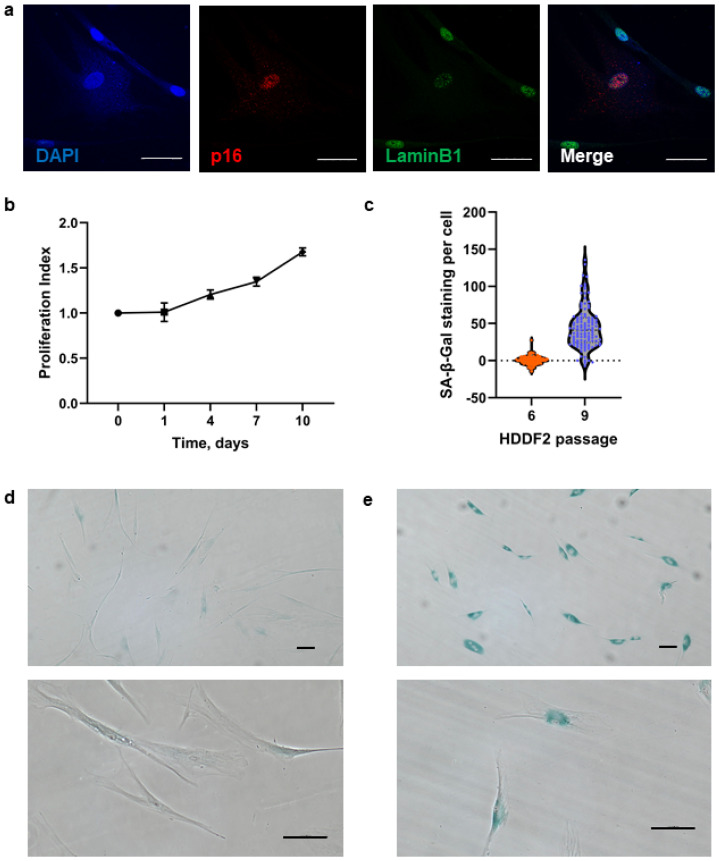
Proliferative characteristics of the HDDF2 dermal fibroblast line: (**a**). Immunofluorescent staining with DAPI dye and senescent-associated proteins p16 and laminB1 illustrating a senescent cell. Confocal microscopy, ×40; scale bar, 50 um. (**b**). The growth curve was represented as a proliferation index, which was calculated as the ratio of the cell number at each time point to the initially plated cell number. The data are shown as mean ± SD. (**c**). SA-β-Gal fluorescence intensity per cell at passage 6 (*n* = 110) and passage 9 (*n* = 115). Cells from three independently seeded dishes were pooled for analysis. Box plots show median and interquartile range; whiskers indicate 10th–90th percentiles. Each dot represents one cell. (**d**). Cells at passage 6 were stained with SA-β-Gal. Light microscopy, ×4 and ×10; scale bar, 100 um. (**e**) Cells at passage 9 were stained with SA-β-Gal. Light microscopy, ×4 and ×10; scale bar, 100 um.

As HDDF2 has demonstrated two subpopulations of cells, further investigation into their senescent nature has been conducted. These cells have stained positively for the senescence-associated marker p16 and shown reduced levels of lamin B1, a molecule associated with cellular senescence (Figure 2a). Next, we examined the proliferation activity of the HDDF2 line. Growth curve analysis revealed a markedly reduced proliferation rate (Figure 2b). The average population doubling time was 234.9 ± 31.6 h, which substantially exceeds the range of 35–50 h typically reported for primary human dermal fibroblasts [43,46], indicating a significantly impaired proliferative capacity. These findings indicate that the HDDF2 fibroblast line contains a senescent cell population, which contributes to its reduced growth potential. In addition, these cells exhibited a rather low cloning efficiency: median 0% (IQR: 0–10%; Q1–Q3). Five out of 12 dishes yielded at least one colony. Thus, the majority of dishes showed no colonies, while those with at least one colony yielded one or two colonies.

Taken together, the HDDF2 cell line appears to consist of two subpopulations of fibroblasts. One of these populations is elongated and proliferating, while the other has exhausted its proliferative capacity. To quantify the senescent fraction over time, we performed SA-β-Gal staining at sequential passages (Figure 2d,e). By passage 6, only a small number of cells were positive for SA-β-Gal. By passage 9, SA-β-Gal fluorescence intensity was elevated compared to passage 6 (median 41.0 [25.4; 56.7] vs. 0.0 [−3.4; 3.0] arbitrary units; *n* = 115 and 110 cells, respectively; Figure 2c), confirming the expansion of the senescent subpopulation with continued culture.

To further define the identity of the HDDF2 line, we analyzed its surface marker expression, differentiation capacity, and genomic stability. The phenotype of HDDF2 cells was further characterized using flow cytometry. At passage 7, HDDF2 cells exhibited high positivity for the main MSC surface markers, including CD44 (99.77% ± 0.24), CD73 (99.28% ± 0.57), CD90 (99.45% ± 0.53), CD105 (97.22% ± 2.67), and HLA-ABC (99.27% ± 0.71). At the same time, the cells were negative for hematopoietic lineage markers, with 98.71% ± 0.93 of cells lacking CD34 expression and 99.44% ± 0.37 lacking HLA-DR expression. This immunophenotype is consistent with both classical mesenchymal stromal cells and dermal fibroblasts.

**Figure 3:**
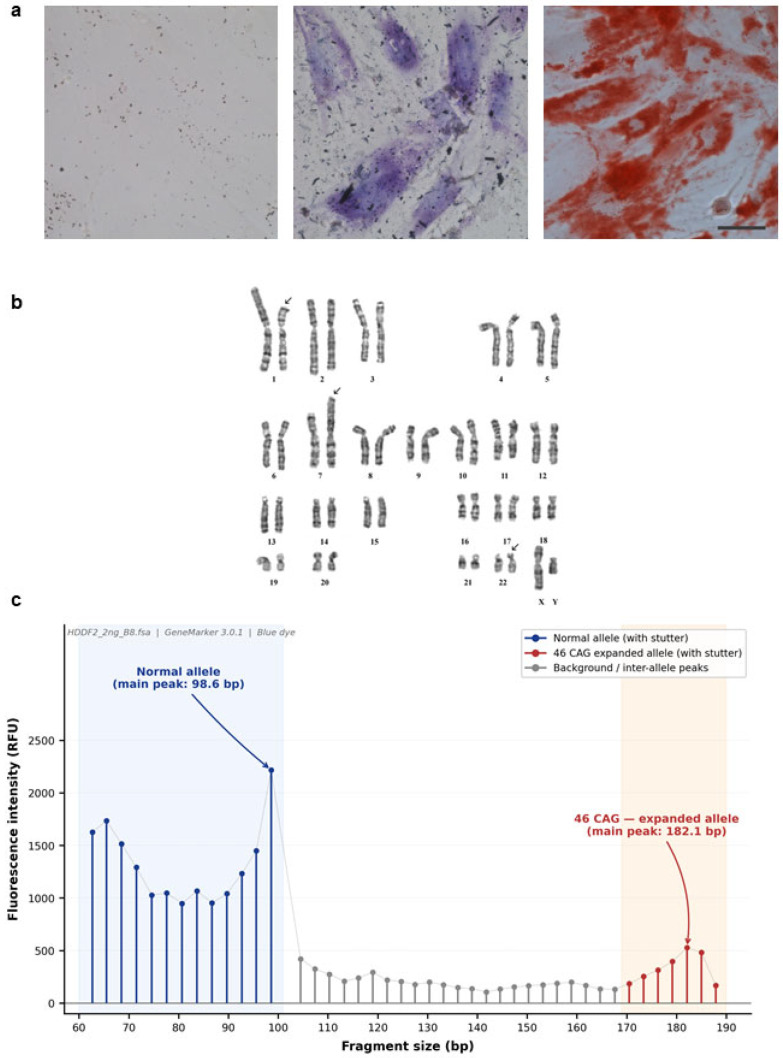
Differentiation potential and karyotype of HDDF2 cells. (**a**). Staining with Oil Red, which stains drops of fats in adipogenic cells; toluidine blue is a marker of chondrogenic differentiation; Alizarin Red detects osteogenic cells due to binding calcium. Light microscopy, ×4; scale bar, 150 um. (**b**). Karyotype of HDDF2 cells with a normal number of chromosomes (46 XY) and a clonal translocation between chromosomes 1 and 7 (marked with an arrow), and non-clonal structural rearrangements involving chromosomes 16, 20, and 22. (**c**) Fragment analysis of the *HTT* CAG repeat region in HDDF2 fibroblasts. Each data point represents a detected peak (fragment size and fluorescence intensity) from capillary electrophoresis of genomic DNA (2 ng input, passage 8). The normal allele (blue) shows a main peak at 98.6 bp with characteristic stutter peaks; the expanded mutant allele (red) shows a main peak at 182.1 bp, corresponding to 46 CAG repeats.

To assess the functional mesenchymal potential suggested by the surface marker profile, we subjected HDDF2 cells to adipogenic, osteogenic, and chondrogenic induction. After three weeks of culture in differentiation media, the cells showed no lipid droplet accumulation (Oil Red O staining), indicating a lack of adipogenic potential under these conditions. However, they demonstrated a clear capacity for osteogenic differentiation, as evidenced by extracellular matrix mineralization (Alizarin Red staining), and for chondrogenic differentiation, shown by the deposition of sulfated glycosaminoglycans (Toluidine Blue staining) (Figure 3a).

Karyotyping of 50 metaphase cells revealed a pseudodiploid karyotype (2n = 46, XY) in the majority of cells. A reciprocal translocation between chromosomes 1 and 7—der(1)t(1;7)(p22;p13) and der(7)t(1;7)(p22;p13)—was present in all analyzed cells at both passages 6 and 8, confirming its clonal stability across passages (Figure 3b). Additionally, non-clonal structural rearrangements involving chromosomes 16, 20, and 22 were observed in a small subset of cells, indicating a degree of karyotypic instability (Figure 3b).

To confirm the disease-relevant genotype of the HDDF2 line, the *HTT* CAG repeat length was verified by fragment analysis of genomic DNA extracted from fibroblasts at passage 8. Capillary electrophoresis revealed two distinct alleles: a normal allele with a main peak at 98.6 bp and an expanded mutant allele with a main peak at 182.1 bp, corresponding to 46 CAG repeats (Figure 3c). The expanded allele was represented by a discrete, well-defined peak cluster with a characteristic stutter pattern, confirming repeat integrity and the absence of detectable somatic mosaicism at this passage.

**Figure 4:**
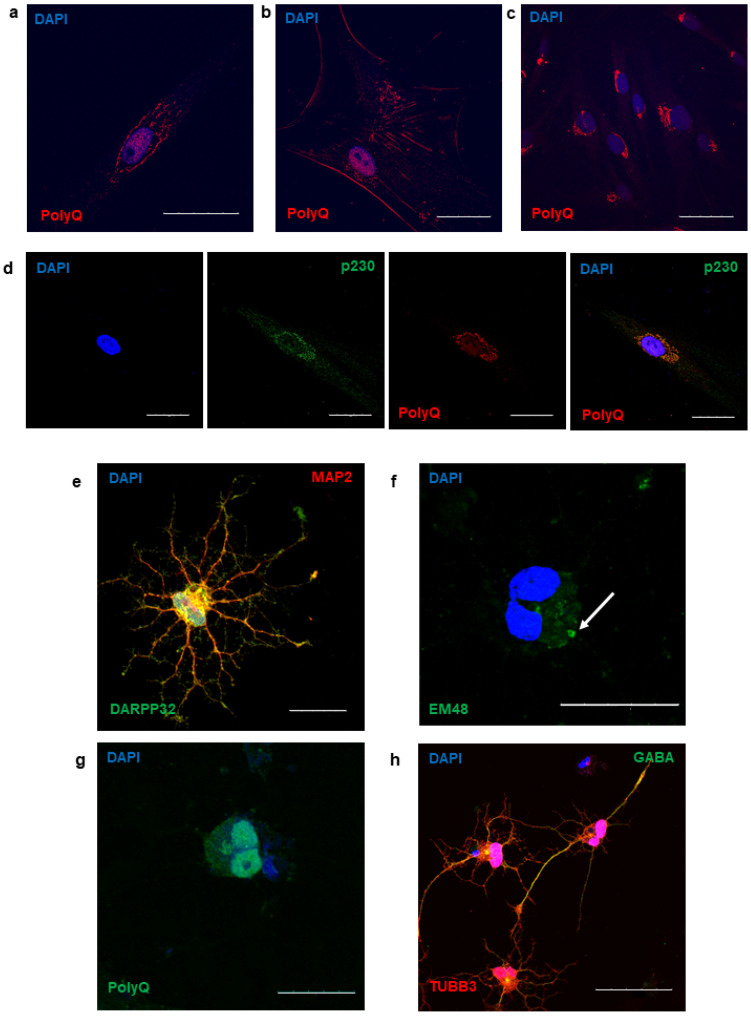
Immunofluorescent staining of elongated, spindle-shaped (**a**) and enlarged subpopulations (**b**) of HDDF2 fibroblasts and control non-HD fibroblasts (**c**) for DAPI and polyQ, and immunofluorescent staining of induced neurons obtained from HDDF2 for DARPP32 and MAP2 (**e**), for EM48 (**f**), for polyQ (**g**), and for GABA and TUBB3 (**h**). (**d**). Immunofluorescent staining of p230 and polyQ. Confocal microscopy, ×60; (**a**–**d**,**h**)—scale bar, 50 um; (**e**–**g**)—scale bar, 20 um.

It is well-established that the presence of a long polyglutamine stretch results in the creation of a mutant protein and the accumulation of aggregates within cells. To investigate this phenomenon, we employed antibodies that specifically detect the polyglutamine sequence—Anti-Polyglutamine-Expansion Diseases Marker Antibody (polyQ)—to stain fibroblasts (Figure 4a). In the elongated, spindle-shaped subpopulation of HDDF2 fibroblasts, polyQ stained in the perinuclear region, morphologically similar in structure to the Golgi apparatus, as confirmed by co-staining with the trans-Golgi marker p230 (Figure 4d); a similar perinuclear pattern was observed in control non-HD fibroblasts (HdFb220, Figure 4c). However, in the subpopulation with large cells (Figure 4b) staining positive for p16, polyQ staining was characterized not only by perinuclear staining, as in the case of spindle-shaped cells, but also by elongated fibrils, which may be due to mHTT association with the cytoskeleton and also protein clusters within the cytoplasm far beyond the perinuclear region (Figure 4a). Finally, we induced HDDF2 reprogramming into striatal neuronal cells—medium spiny neurons—according to the protocol described by Richner M. et al. [40] with further modifications as previously described [45]. The fibroblasts of the HDDF2 lineage successfully transformed into neurons, as evidenced by the staining of the resulting cells with the neuronal markers TUBB3 (tubulin-beta III), GABA, DARPP32, and MAP2 (Figure 4e–h).

## 4. Discussion

Human patient-derived cell models are essential to bridge the translational gap in HD research. Dermal fibroblasts represent one of the most readily available patient-derived biomaterials and, due to their characteristics, serve as a fundamental cellular component for the development of disease models. The procedure for extracting this biomaterial (punch biopsy) is minimally invasive and performed under local anesthesia, without impairing the patient’s functional capacity. The resulting tissue fragment is typically no larger than 4 mm in diameter, yet it allows for the generation of a culture of actively proliferating dermal fibroblasts that can maintain growth for an extended period (up to 50 population doublings), while preserving the essential properties of patient-specific somatic cells [43,47]. In this study, we established and characterized a novel dermal fibroblast line, HDDF2, from a patient with HD to model age-related features in cells in culture. The cell line is available in the Russian Type Culture Collection (RTCC)—https://cellcollection.ru/catalog/hddf2/ (accessed on 31 May 2026). Dermal fibroblasts generally exhibit the immunophenotypic characteristics of mesenchymal stem cells and have the potential to differentiate along osteogenic, chondrogenic, and adipogenic pathways when cultured in appropriate, specialized media. However, individual lines may differ in basic cellular characteristics, including proliferative activity, population doubling time, and differentiation potential. Additionally, there are differences in their ability to demonstrate stem cell properties, including their capacity to differentiate into various types of cells and tissue derivatives. These differences may be due to both the individual characteristics of the donor, such as the patient’s age, gender, and the presence of certain diseases, and the methodology used for culture establishment. The isolation of cells from the body can lead to the acquisition of somatic mutations, which may then be consolidated within the cell population. Additionally, the conditions under which the cells are cultured can promote the selection and subsequent growth of only a subset of cells. Based on this, each newly established primary cell culture must be thoroughly characterized, both in terms of the individual characteristics of the donor and the fundamental characteristics and properties of the cell line itself [41,42,48,49]. This information is essential for determining the potential use of a cell line in creating disease models, generating accurate samples of cell lines for research, and other purposes.

Throughout cultivation, the HDDF2 line consistently comprised two distinct subpopulations: actively proliferating fibroblasts and cells entering replicative senescence. The proliferating subpopulation maintained the typical spindle-shaped morphology and supported serial passaging; meanwhile, the proportion of senescent cells progressively increased with continued culture. By passage 9, senescent cells became increasingly prominent, characterized by an enlarged, flattened morphology and positive staining for senescence-associated β-galactosidase (SA-β-Gal). Immunofluorescence analysis confirmed their senescent identity, revealing increased expression of the cell cycle inhibitor p16 and reduced levels of the nuclear envelope protein lamin B1—both were established hallmarks of replicative senescence. The presence of this senescent subpopulation from early passages likely contributes to the overall reduced proliferative capacity of the HDDF2 line, as evidenced by its prolonged population doubling time. The pronounced senescence-associated phenotype observed in the HDDF2 line contrasts with the robust proliferation reported for the fibroblast line HDDF derived from a female HD patient with a similar total CAG repeat length [50]. A potential explanation is that the line was derived from a male donor with advanced-stage HD. Although cross-sectional studies have shown a faster progression of the disease in men [51], other data suggest a different correlation, indicating a more severe and rapid progression in female patients [52,53]. Based on the available literature, these data suggest that HD may have gender-specific manifestations and rates of progression. This variability highlights the inherent heterogeneity in patient-derived cell models. While donor-specific factors such as sex or disease stage may contribute, an alternative explanation also could involve differences at the DNA level. Recent evidence indicates that the pathogenic driver is not the total polyQ tract length, but rather the length of the uninterrupted CAG repeat tract, with CAA interruptions that mitigate toxicity [54,55].

Despite the clear signs of replicative senescence, the cells maintained the immunophenotype of mesenchymal stromal cells (MSCs). These findings are in agreement with previous studies on the generation and characterization of MSC lines derived from skin and other tissues. These studies have shown that the MSC phenotype remains stable even after several passages, before complete loss of cell proliferation [41,42,48,49]. Additionally, the reduced differentiation capacity in the culture of aged cells, as evidenced by the lack of adipogenesis following induction, is also consistent with previously reported data [47,56].

Karyotypic analysis of the HDDF2 cell line revealed two distinct classes of chromosomal alteration. First, a balanced translocation between chromosomes 1 and 7 (t(1;7)(p22;p13)) was present in all 50 metaphases analyzed. However, the origin of this translocation cannot be confirmed without karyotyping the donor’s peripheral blood lymphocytes, which was not feasible in this study. The breakpoints at 1p22 and 7p13 do not overlap with known loci involved in HD pathogenesis; this translocation is therefore unlikely to be related to HD pathogenesis. Researchers using the HDDF2 line should be aware of this finding and consider its possible effects on experimental results.

Second, non-clonal structural rearrangements of chromosomes 16, 20, and 22 were detected in a small proportion of cells. Unlike the clonal t(1;7), these rearrangements are more likely to be caused by ongoing somatic karyotypic instability, rather than representing a constitutionally inherited alteration. Growing evidence suggests that mutant huntingtin can interfere with DNA repair pathways and promote genomic instability [50,57], raising the possibility that these non-clonal rearrangements reflect elevated chromosomal fragility in HD patient-derived cells. The HDDF2 cell line may therefore be useful for investigating the relationship between huntingtin and genomic integrity in non-neuronal cells.

Notably, HDDF2 fibroblasts retain a capacity for neuronal conversion, as demonstrated by direct reprogramming into TUBB3-, GABA-, and DARPP-32-positive induced neurons. In fibroblasts, we observed cytoplasmic polyQ inclusions, particularly in the senescent subpopulation, which is consistent with the data on the early involvement of the secretory pathway and the Golgi apparatus in the pathogenesis of HD. In contrast, in induced striatal neurons 40 days after reprogramming, anti-polyQ antibodies revealed predominantly diffuse nuclear staining. Diffuse nuclear accumulation of mutant huntingtin may precede the formation of insoluble intranuclear inclusions. The relatively short neuronal culture period (40 days) relative to the protracted disease course in vivo may explain why this stage is observed at this time point. This is consistent with reports from our group and others describing cytoplasmic polyQ aggregate detection [58,59]. These findings indicate that disease-relevant cellular pathology is recapitulated in the HDDF2 line. Therefore, the HDDF2 cell line is a valuable cellular resource for studying age-related features associated with this disease. The HDDF2 cell line, together with similar lines from patients of different ages, may serve as a starting material for future neuronal reprogramming studies of the molecular mechanisms of age-related HD pathogenesis and the influence of donor-specific factors on the observed cellular phenotype.

Several limitations of the present study should be acknowledged. First, HDDF2 represents a single patient-derived line established without a matched healthy-donor control or a parallel HD line; conclusions regarding HD-associated cellular phenotypes therefore cannot be generalized beyond this donor, and all observed features should be interpreted as characteristics of this specific line. Second, the origin of the clonal t(1;7)(p22;p13) translocation—whether constitutional or culture-acquired—remains unresolved, as karyotyping of donor peripheral blood lymphocytes was not feasible; potential phenotypic consequences of this rearrangement for experimental results cannot be excluded.

## Figures and Tables

**Table 1:** Antibodies used for flow cytometry.

Antibody	Vendor	Catalog Number
Iso PE	BD Pharmingen (Milpitas, CA, USA)	554680
CD90 PE	BD Pharmingen (Milpitas, CA, USA)	561970
CD73 PE	BD Pharmingen (Milpitas, CA, USA)	550257
CD105 PE	BD Pharmingen (Milpitas, CA, USA)	560839
CD44 PE	eBioscience (San Diego, CA, USA)	12-0441-81
CD34 PE	Beckman Coulter (Brea, CA, USA)	IM1420
Iso FITC	BD Pharmingen (Milpitas, CA, USA)	555748
HLA-ABC FITC	Beckman Coulter (Brea, CA, USA)	IM1838U

## Data Availability

The raw quantitative data supporting the results of this study are openly available in Zenodo at https://doi.org/10.5281/zenodo.20518328. Further inquiries can be directed to the corresponding author(s).

## Abbreviations

The following abbreviations are used in this manuscript:

polyQ	Polyglutamine
HD	Huntington’s disease
iPSC	Induced pluripotent stem cell
MSN	Medium spiny neuron
STR	Short tandem repeat
SA-β-gal	Senescence-associated β-galactosidase
MSC	Mesenchymal stromal cell
